# A New Approach for Evaluating Electron Transfer Dynamics by Using *In Situ* Resonance Raman Microscopy and Chronoamperometry in Conjunction with a Dynamic Model

**DOI:** 10.1128/AEM.01535-20

**Published:** 2020-10-01

**Authors:** Adolf Krige, Kerstin Ramser, Magnus Sjöblom, Paul Christakopoulos, Ulrika Rova

**Affiliations:** a Biochemical Process Engineering, Division of Chemical Engineering, Department of Civil, Environmental and Natural Resources Engineering, Luleå University of Technology, Luleå, Sweden; b Experimental Mechanics, Division of Fluid and Experimental Mechanics, Department of Engineering Sciences and Mathematics, Luleå University of Technology, Luleå, Sweden; North Carolina State University

**Keywords:** online resonance Raman, chronoamperometry, electron transfer, *Geobacter sulfurreducens*, dynamics, bioelectrochemical system, OmcS

## Abstract

Geobacter sulfurreducens is a good candidate as a chassis organism due to its ability to form thick, conductive biofilms, enabling long-distance extracellular electron transfer (EET). Due to the complexity of EET pathways in G. sulfurreducens, a dynamic approach is required to study genetically modified EET rates in the biofilm. By coupling online resonance Raman microscopy with chronoamperometry, we were able to observe the dynamic discharge response in the biofilm’s cytochromes to an increase in anode voltage. Measuring the heme redox state alongside the current allows for the fitting of a dynamic model using the current response and a subsequent validation of the model via the value of a reduced cytochrome *c* Raman peak. The modeled reduced cytochromes closely fitted the Raman response data from the G. sulfurreducens wild-type strain, showing the oxidation of heme groups in cytochromes until a new steady state was achieved. Furthermore, the use of a dynamic model also allows for the calculation of internal rates, such as acetate and NADH consumption rates. The Raman response of a mutant lacking OmcS showed a higher initial oxidation rate than predicted, followed by an almost linear decrease of the reduced mediators. The increased initial rate could be attributed to an increase in biofilm conductivity, previously observed in biofilms lacking OmcS. One explanation for this is that OmcS acts as a conduit between cytochromes; therefore, deleting the gene restricts the rate of electron transfer to the extracellular matrix. This could, however, be modeled assuming a linear oxidation rate of intercellular mediators.

**IMPORTANCE** Bioelectrochemical systems can fill a vast array of application niches, due to the control of redox reactions that it offers. Although native microorganisms are preferred for applications such as bioremediation, more control is required for applications such as biosensors or biocomputing. The development of a chassis organism, in which the EET is well defined and readily controllable, is therefore essential. The combined approach in this work offers a unique way of monitoring and describing the reaction kinetics of a G. sulfurreducens biofilm, as well as offering a dynamic model that can be used in conjunction with applications such as biosensors.

## Abstract (precis)

Bioelectrochemical systems can fill a vast array of application niches, due to the control of redox reactions that it offers. Although native microorganisms are preferred for applications such as bioremediation, more control is required for applications such as biosensors or biocomputing. The development of a chassis organism, in which the EET is well defined and readily controllable, is therefore essential. The combined approach in this work offers a unique way of monitoring and describing the reaction kinetics of a G. sulfurreducens biofilm, as well as offering a dynamic model that can be used in conjunction with applications such as biosensors.

## INTRODUCTION

Since the discovery of extracellular electron transfer (EET), many microorganisms have been reported as being electroactive, with a surprising level of diversity in mechanisms, pathways, and species (1). Of these, Geobacter sulfurreducens remains intensely studied, due to its ability to form thick, conductive biofilms and to generate high current densities in microbial fuel cells (MFCs) (2). G. sulfurreducens was found to have highly conductive pili, which enable efficient long-distance EET and formation of thick biofilms (3). This makes G. sulfurreducens a good candidate to serve as a chassis microorganism for the study of mechanisms involved in distance EET. However, the pathways involved in the EET in G. sulfurreducens are complex and multiple pathways have been described, with formal potentials of the pathways ranging from −0.1 V to −0.25 V versus a standard hydrogen electrode (SHE) (4).

The genome of G. sulfurreducens genome contains 111 putative genes coding for *c*-type cytochromes. The abundance of cytochromes allows G. sulfurreducens to efficiently adapt to disruptions of an electron transfer pathway, which makes it significantly more difficult to interpret the impact of a deleted gene (5). This is evident from the change in opinion regarding the role of the outer membrane cytochrome S (OmcS). OmcS was initially believed to play a role in the conductivity of the electrically conductive pili present in G. sulfurreducens (6). However, this has been contradicted by several studies, in which high currents have been observed in strains lacking OmcS and increased biofilm conductivity has even been obtained after deleting OmcS (7–9). Recently, some progress has been made in establishing the minimum requirements for EET in G. sulfurreducens. Yet it was also shown that deleting a gene of interest can sometimes have unexpected consequences (10). A better understanding of the EET mechanisms, including how the pathways adapt and knowledge on possible negative impacts of deleting various pathways, is needed if G. sulfurreducens is to be used as a chassis for specialized bioelectrochemical applications (10).

**FIG 1:**
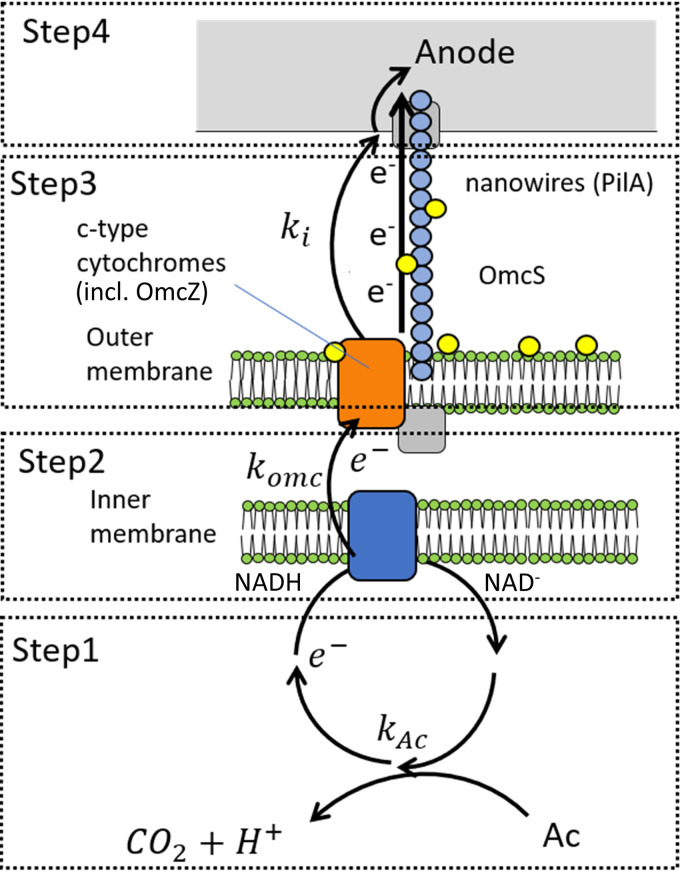
Schematic presentation of the redox reactions involved in the EET in a biofilm for the proposed reaction scheme along with the rate constants of steps 1 to 4 (*k*_Ac_, *k*_Omc_, *k*_cond_, and *k_i_*, respectively).

Even though the complete mechanism of EET is quite complicated, a simplified reaction scheme for describing EET in a biofilm of G. sulfurreducens can be used to simulate the reaction kinetics. In the model, the EET within a biofilm is essentially split into four redox steps, as shown schematically in Fig. 1. This is based on a similar scheme used for modeling cyclic voltammetry data (11), which, in turn, was based on classic catalyst-dependent electrode reactions, assuming an electrode-bound, nondiffusing catalyst with the electron transfer done via reduction of electrode-reactive mediators (12, 13).

The different electrochemical reactions taking place in each step in G. sulfurreducens can be expressed by the following reaction scheme:

$$1.\frac{1}{8}{\text{Ac}}^{-}+{\text{IM}}_{\text{ox}}\overset{k_{\text{Ac}}}{\to }{\text{IM}}_{\text{red}}+\frac{1}{4}{\text{CO}}_{\text{2}}{\text{+H}}^{\text{+}}$$

$$\text{2}{\text{. IM}}_{\text{red}}{\text{+ Omc}}_{\text{ox}}^{d=d_{0}}\overset{k_{\text{Omc}}}{\to }{\text{IM}}_{\text{ox}}+{\text{Omc}}_{\text{red}}^{d=d_{0}}$$

$$\text{3}{\text{. Omc}}_{\text{red}}^{d=d_{0}}+{\text{Omc}}_{\text{ox}}^{d=0}\overset{k_{\mathrm{cond}}}{\to }{\text{Omc}}_{\text{ox}}^{d=d_{0}}+{\text{Omc}}_{\text{red}}^{d=0}$$

$$\text{4}{\text{. Omc}}_{\text{red}}^{d=0}\overset{k_{\text{cond}}}{\to }{\text{Omc}}_{\text{ox}}^{d=0}+e_{\text{electrode surface}}^{-}$$

The first step in the reaction scheme is the oxidation of the main substrate, acetate (Ac^–^) in this case, via the cellular metabolism, and the reduction of an internal mediator (IM_red_) with a rate constant *k*_Ac_, generating carbon dioxide and protons as by-products. During normal operation of an MFC, this is expected to be the rate-limiting step, i.e., the current is limited by the metabolic conversion of the substrate (11). This would mean that *k*_Ac_ should be smaller than the other rate constants during normal operation. It has also been found that if acetate is above a certain concentration (approximately 5 mM), the rate of acetate consumption is considered to be only a function of IM_red_ and independent of the acetate concentration.

The second step involves the intracellular electron transfer across the cell membrane, with a rate constant *k*_Omc_, resulting in the reduction of cell-bound outer membrane cytochromes (Omc_ox_) and the subsequent oxidation of the internal mediator (IM_ox_). This step is also metabolically beneficial for the microbe, consuming NADH and pumping H^+^ across the inner membrane, which, in turn, can be used to produce ATP, as seen in Fig. 1.

Step 3 is the long-distance electron transport through the biofilm of G. sulfurreducens, transferring electrons from mediators at the cell surface (${\text{Omc}}_{\text{red}}^{d=d_{0}}$, at distance *d* = *d*_0_) to mediators at the anode surface (${\text{Omc}}_{\text{red}}^{d=0}$, at *d* = 0). Richter et al. (11) have previously proposed a model in which mediators are fixed in the extracellular domain of the biofilm and EET occurs via a series of electron transfer reactions among adjacent ET mediators. However, there have been several studies showing that long-range electron transfer occurs not via electron transfer between adjacent *c*-type cytochromes (via electron hopping/tunneling) but rather via “e-pili” possessing delocalized electronic states, essentially functioning as metallic-like protein wires (14, 15).

In step 4, interfacial electron transfer, mediators at the electrode surface (${\text{Omc}}_{\text{red}}^{d=0}$) transfer electrons to the electrode, with an interfacial rate constant of *k_i_*, producing a current. The rate constant of the electron transfer to the anode (*k_i_*) depends on the potential at which the anode is poised (*E*), which can be described by Butler-Volmer electrode reaction rate expression (16). In Butler-Volmer expression, if the potential is sufficiently higher than the formal potential of the mediator (*E*^0^), the anodic reaction will dominate the cathodic. The simplified rate constant equation can then be expressed as equation 1:

(1) $$k_{i}=k^{0}\text{exp}\left[\left(1-\alpha \right)\left(\frac{\mathrm{nF}}{\mathrm{RT}}\right)\left(E-E^{0}\right)\right]$$

where *E*^0^ is the formal potential of the mediator, *n* is the number of electrons transferred, *k*^0^ is the standard rate constant, α is the transfer coefficient, *T* is temperature (kelvin), *R* is the idea gas constant, and *F* is the Faraday constant (with a value of 8.314…C·V·K^−1^ mol^−1^ and 96,485.3…C·mol^−1^, respectively).

Cyclic voltammetry (CV) is useful for determining the formal potentials of redox couples involved in EET pathways (17), but it suffers from the fact that heterogeneous dynamics can affect the observed response, complicating the extraction of accurate rate constants. Potential step methods, such as chronoamperometry, do not have this problem, since the potential is stepped to values where the heterogeneous reaction is mass transfer controlled (16). Chronoamperometry therefore offers a simpler solution for fitting the rate constants in a model describing the EET in a biofilm. The analysis of the rate constants involved in EET can help to identify rate-limiting factors in various EET pathways. Biofilms of G. sulfurreducens contain many *c*-type cytochromes that are known to be good Raman scatterers. Raman microscopy has shown to be capable of obtaining online biochemical information for G. sulfurreducens biofilms, specifically on the redox state of *c*-type cytochromes, in a nondestructive manner (18). By using short integration times, a time series can be created, showing the dynamic response to a change in environment. This offers the ability of observing the dynamics of the change in redox state of the heme groups using online resonance Raman spectroscopy (19). The steady-state operation and the nondestructive nature of resonance Raman microscopy allow for repeated measurements of intracellular compounds on a single biofilm, as well as changing the biofilm conditions. Since the intensity of the signal of reduced cytochromes is significantly higher than that of oxidized cytochromes, the resonance Raman spectra can be linked to the model by comparing the change in concentration of the reduced cytochromes in the model to the change in the Raman spectra (18). This should show whether the model is a good approximation of the EET mechanisms. While linking Raman spectroscopy and electrochemistry is not new, this has only been done using CV (19).

The aim of this study was to use a modified electron transfer dynamic model in conjunction with resonance Raman microscopy and chronoamperometry to describe the EET rates of a G. sulfurreducens biofilm. The dynamic model was further used to calculate internal rates of acetate and NADH consumption. The resonance Raman data were compared to data for an OmcS-deficient strain in order to show and discuss the effects of altering the EET pathway and to propose a modified dynamic model.

## RESULTS AND DISCUSSION

### Dynamic response and fitting.

By poising the anode at a sufficiently negative value, the rate constant of electron transfer at the surface of the electrode is restricted, as described by Butler-Volmer expression. This causes an increase in the fraction of outer membrane cytochromes in a reduced state, i.e., a buildup of electrons in the biofilm matrix. The cytochromes have been shown to function as capacitors for the cells, storing electrons under some environmental conditions (20, 21). When the poised voltage is then stepped up, there is a spike in the measured current that is much higher than the steady-state current (can be more than 30 A/m^2^). The sampling rate was set as 10 samples per second, and since this peak is maintained for an extremely short time (only one sample), the observed current might not reflect the true maximum current. After the peak the current decreases exponentially to a steady-state value after approximately 100 s (∼0.7 A/m^2^ in this case).

**FIG 2:**
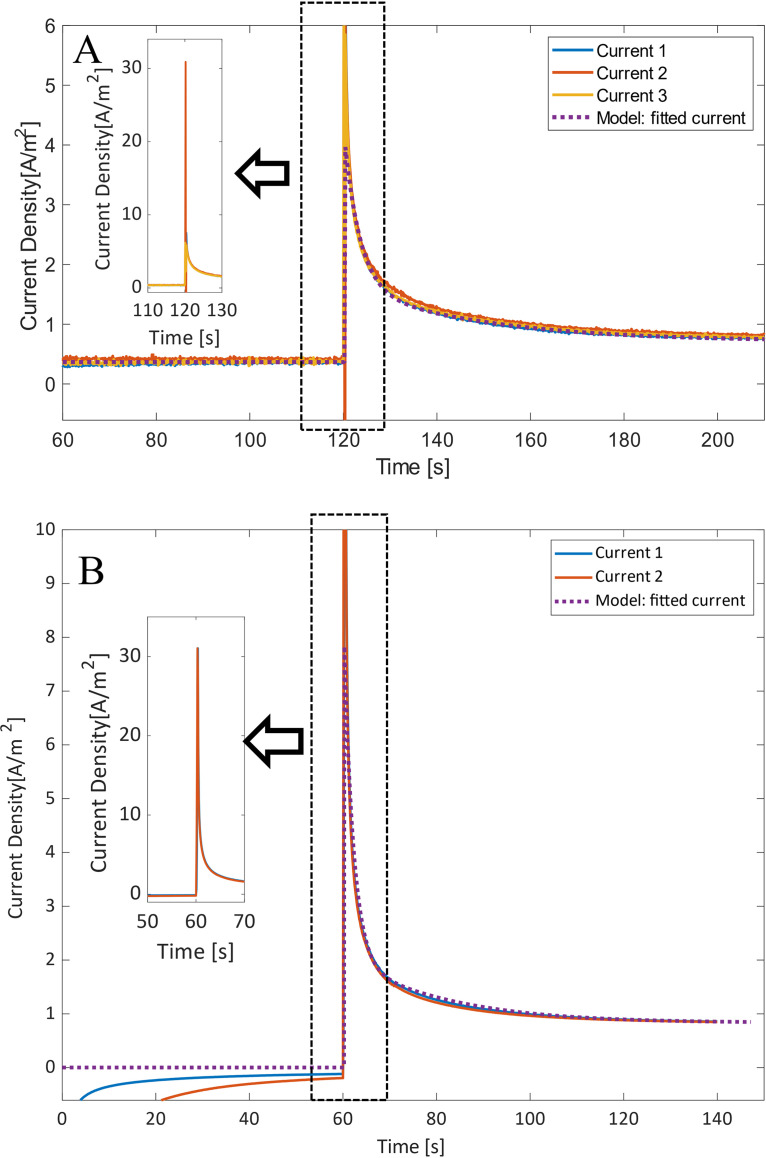
The fitted response of current density per electrode surface area of wild-type G. sulfurreducens biofilm during 2 step changes of poised voltage (WT Δ*E*1, from −101 mV to 499 mV [A], and WT Δ*E*2, from −251 mV to 649 mV [B]).

**FIG 3:**
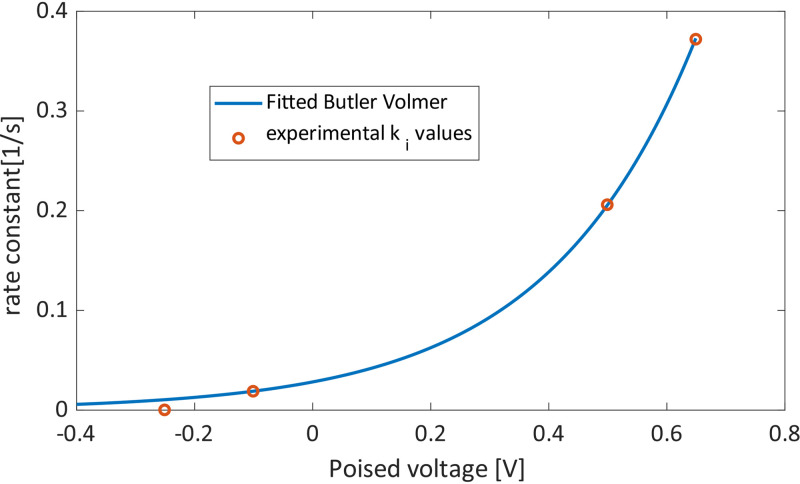
A fitting of the Butler-Volmer equation to the experimentally obtained interfacial rate constant *k_i_*.

The current response from two different poise ranges using the wild-type (WT) strain, i.e., WT Δ*E*1 and WT Δ*E*2 (from −101 mV to 499 mV and from −251 mV to 649 mV, respectively; showing three and two measurements, respectively), can be seen in Fig. 2A and B, respectively. The step timing was adjusted for Δ*E*2 since 60 s was considered enough time to reach a steady state and prolonged time at −251 mV might be detrimental for the biofilm, resulting in metabolic changes. The two sets of poising values used can be seen in Table 1 and Fig. 2. These currents were fitted using the fminsearch function in MATLAB, with the resulting constants shown in Table 1. The chronoamperometric step from −101 mV is above the redox potentials of OmcZ (an outer surface cytochrome essential for optimal current production [22]) and OmcS, at −220 mV and −212 mV, respectively (4, 23), while the other is below their redox potentials, at −251 mV. The step size was also increased from 600 mV to 900 mV. This results in an steady-state current density that is positive at −101 mV and negative at −251 mV (Fig. 2A and B, respectively). The negative current is due to the cathodic reaction rate being slightly higher than the anodic reaction rate, as described by the Butler-Volmer expressions. This can also be seen in Fig. 3, in which the first *k_i_* value is slightly lower than that predicted by the fitting of the anodic Butler-Volmer expression.

**TABLE 1:** Best-fit constant values for the response of G. sulfurreducens at different voltages as well as that of an OmcS-deficient mutant (WT Δ*E*1, WT Δ*E*2, and ΔOmcS, respectively)

Parameter	Value for indicated strain		
	WT Δ*E*1	WT Δ*E*2	ΔOmcS
Voltage min (mV)	−101	−251	−251
Voltage max (mV)	499	649	649
${\text{[IM]}}^{\text{t}}{}^{\text{ot}}$ (nmol)	2,555	2,216	2,908
${\text{[Omc]}}^{\text{t}}{}^{\text{ot}}$ (nmol)	814	723	611
*k*_Ac_ (nmol)	1.33E−02	2.17E−02	2.11E−02
*k*_Omc_ (nmol)	4.99E−05	4.58E−05	5.01E−05
*k*_Omc_ × [Omc]^tot^ (1/s)	4.06E−02	3.31E−02	3.06E−02
$k_{i}^{\text{bef}}$ (1/s)	1.89E−02	2.26E−04	4.61E−03
$k_{i}^{\text{aft}}$ (1/s)	2.06E−01	3.72E−01	3.16E−01

**FIG 4:**
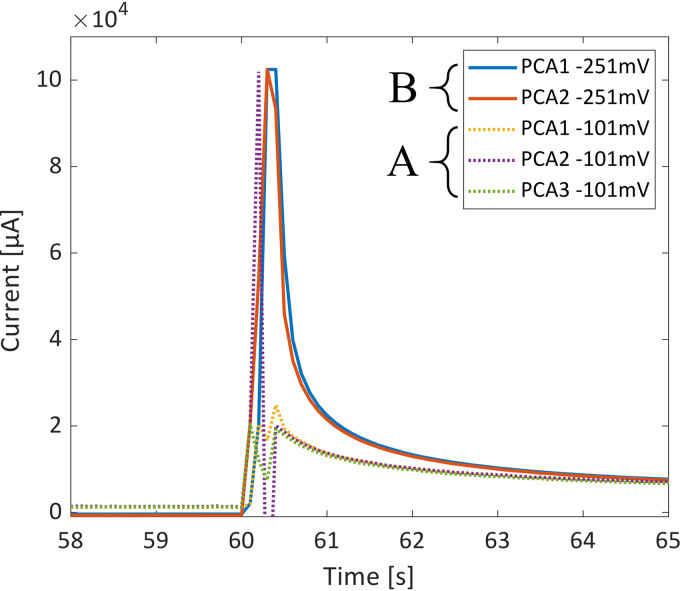
Detailed view of the response of current density per electrode surface area of G. sulfurreducens biofilm, during 2 step changes of poised voltage; the timing was adjusted to align the change in potential (WT Δ*E*1, from −101 mV to 499 mV [A], and WT Δ*E*2, from −251 mV to 649 mV [B]).

The current densities look quite similar at the scale of Fig. 2; however, Fig. 4 shows a detailed view, in which the difference in slope and maximum current obtained can be seen. The increase in maximum current in wild-type test 2 is due to a larger fraction of the cytochromes that are initially reduced, since a lower initial voltage is used, as well as the final voltage being higher, resulting in a larger $k_{i}^{\text{aft}}$. Here it is also possible to see an initial sharp decrease of the current in the samples poised at −101 mV (current 2 [Fig. 2] reaches −12 A/m^2^). It is not clear what causes this, yet this lasts only 0.3 s, i.e., only 3 sample points, so it is possible that this is simply an artifact of the measurement system used and a high-sample-rate potentiostat would be needed to test this phenomenon.

From Table 1, for Δ*E*1, one can see that $k_{i,\Delta E1}^{\text{bef}}$ and *k*_Ac, Δ_*_E_*_1_ have similar values; however, $k_{i,\Delta E1}^{\text{aft}}$ increases to more than 10 times $k_{i,\Delta E1}^{\text{bef}}$, and it is this change that causes the sharp spike in the observed current. When comparing Δ*E*1 to Δ*E*2, on the other hand, one can see that the only value that was drastically changed was $k_{i}^{\text{bef}}$, i.e., $k_{i,\Delta E2}^{\text{bef}}$, which is more than 100 times smaller than $k_{i,\Delta E1}^{\text{bef}}$. This drastic change was expected and is reflected in the Butler-Volmer equation. This is because the more negative voltage change would result in a sign change in the exponent factor (*E* – *E*^0^) of equation 1 (*E*^0^ is expected to be close to the formal redox potential of OmcS at −212 mV). The change in $k_{i}^{\text{aft}}$ due to the different final poised voltage (i.e., 499 and 649 mV) is not nearly as significant, yet there is an increase, as expected. This change in *k_i_* is visualized in Fig. 3, in which *k_i_* is fitted to the Butler-Volmer equation.

*k*_Omc_ is significantly lower than the other rate constants because it is a second-order reaction. In order to compare *k*_Omc_ with the other rate constants, we multiplied it by the total concentration of extracellular mediations (*k*_Omc_ × [Omc]^tot^). Since most of the extracellular mediations should be oxidized if *E* ≫ *E*^0^ (i.e., [Omc]^tot^ ∼ [Omc_ox_]), this should give a value that can be related to the other rate constants. Since *k*_Omc_ × [Omc]^tot^ is significantly higher than *k*_Ac_ in all fittings, it can be assumed that *k*_Ac_ is the limiting rate constant at high E values. This fits with what was expected, that the oxidation of Ac is the rate-limiting step.

**FIG 5:**
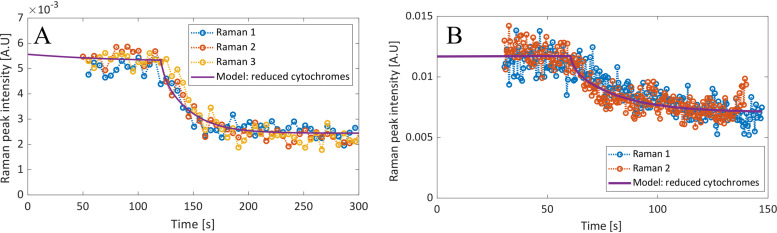
The Raman peak intensity response of an MFC cell, during 2 step changes of poised (WT Δ*E*1, from −101 mV to 499 mV [A], and WT Δ*E*2, from −251 mV to 649 mV [B]) with the total reduced cytochromes from the model.

Since resonance Raman spectroscopy cannot distinguish between the extracellular and intracellular cytochromes, the change in the 747-cm^−1^ Raman peak (related to cytochrome oxidation state) was compared to the sum of the reduced mediators (i.e., [IM_red_] + [Omc_red_]). The Raman data essentially represent the integral of the current graph. The current reflects change in the electrons lost per second (C/s/m^2^) due to the oxidation of the cytochromes, whereas the Raman data reflect the total electrons lost (related to C/m^2^). Therefore, the Raman data showed not an initial spike but rather a sudden decrease in slope, followed by a steady increase in the slope until a new steady-state value was obtained, as seen in Fig. 5. The Raman data matched the shape of the modeled reduced mediators well, even at different poised voltages, showing that the rate-limiting factors of the EET can be effectively described by the simplified model.

**FIG 6:**
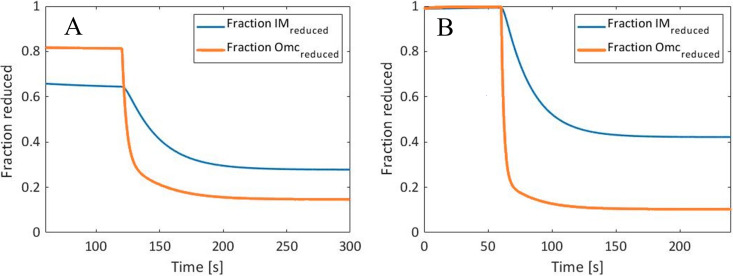
Modeled mediator concentrations, displayed as a fraction of the total available (WT Δ*E*1, from −101 mV to 499 mV [A], and WT Δ*E*2, from −251 mV to 649 mV [B]).

Although the normalized Raman response in Fig. 5 looks very similar, the difference between the modeled reduced cytochromes for the two step sizes are significantly more apparent when observed as the modeled fraction of [Omc]^tot^ and [IM]^tot^, as seen in Fig. 6. For WT Δ*E*1 (at −101 mV), only 65 to 82% of the cytochromes were in a reduced state prior to the step change, whereas in WT Δ*E*2 (at −251 mV), all the cytochromes were essentially in a reduced state. There is also a larger difference between the final steady-state values of [Omc]^red^ and [IM]^red^ in Δ*E*2 (at −649 mV) due to the larger difference between $k_{i}^{\text{bef}}$ and $k_{i}^{\text{aft}}$.

The results show that a transient current profile of a G. sulfurreducens wild-type biofilm, during a step change of poised voltage, could be sufficiently modeled using a simplified reaction scheme. This held true even when different step voltages were used (from −101 mV to 499 mV and from −251 mV to 649 mV). This allowed for the estimation of rate constants describing the dynamics involved in EET. The model was further verified by resonance Raman microscopy by comparing the fitted response of the current density with the Raman peak area, representing the oxidation state of the *c*-type cytochromes. When the peak area was compared to the total reduced mediator concentration calculated from the model, they showed an extremely strong correlation.

Bonanni et al. used a similar approach to fit the discharge of a G. sulfurreducens biofilm by disconnecting the anode and reconnecting it after a period (24). However, instead of simply using the rates of the reoxidation of interfacial cytochromes at the electrode, the current was calculated as the sum of the rates of the reoxidation of interfacial cytochromes, the reoxidation of matrix cytochromes, and the reoxidation of internal cytochromes caused by acetate metabolism. Although this resulted in a fair approximation of the current, it does not account for the interdependence of the rate equations; for example, the interfacial current is simply modeled as an exponential decline of the initial reduced cytochromes [*I*_int_ = *k*_int_Omc_red0_ exp(−*k*_int_*t*)]. This means that the interfacial current is effectively zero, seconds after the start of the discharge, even though a current is still measured. In the modified model presented in this paper, the interdependence of all the rates are considered.

### Determining Butler-Volmer constants.

In order to estimate the formal potential of the ET mediator (*E^0^*′) in the final electron transfer step, the Butler-Volmer equation was fitted to the *k_i_* values (Table 1). The fitted *k_i_* values before and after were used to fit *k*_0_, α, and *E*^0^, giving values of 0.0131 s^−1^ and 0.897 and −0.193 V, respectively, which are slightly above the standard potential of OmcZ, believed to be crucial for EET under low electrode potential (4). Although the Butler-Volmer equation described the data well (Fig. 3), the *k_i_* at −251 mV is lower than expected, which is due to the cathodic reaction becoming sufficiently large, resulting in a negative current.

### Metabolic implications of the rate model.

**FIG 7:**
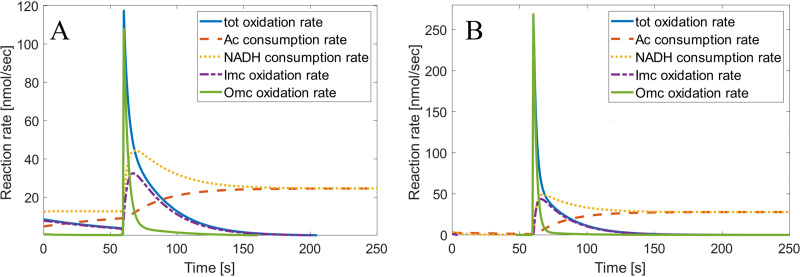
The NADH and acetate consumption rate alongside mediator oxidation rates (WT Δ*E*1, from −101 mV to 499 mV [A], and WT Δ*E*2, from −251 mV to 649 mV [B]).

The model can be linked to the NADH and acetate consumption rate, as seen in Fig. 7. From this, one can see the large difference in the maximum [Omc]^red^ oxidation rate (108 nmol/s, compared to 268 nmol/s) due to the difference in poise values. Furthermore, one can see that the NADH consumption rate is the [Imc]^red^ oxidation rate plus the acetate consumption rate, with the NADH consumption rate approaching the acetate consumption rate as steady state is achieved.

### Comparison with OmcS-deficient strain.

**FIG 8:**
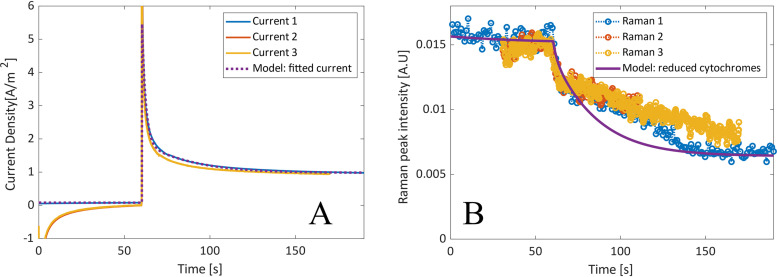
The fitted response of current density per electrode surface area (A) and Raman peak intensity (B) of an OmcS-deficient biofilm during a step change of poised voltage (from −251 mV to 649 mV).

The same experiment was conducted using an OmcS-deficient mutant, using a step in poised voltage from −251 mV to 649 mV (Fig. 8). The fitted variables were very similar to that of the wild type at the same poised values (WT Δ*E*2), with the only significant difference being that $k_{i,\text{OmcS}}^{\text{bef}}$ was more than 20 times larger than when the same voltages were used for G. sulfurreducens. This implies that an alternative pathway, which does not involve OmcS, is utilized.

$$k_{i,\text{step 2}}^{\text{bef}}<k_{i,\text{OmcS}}^{\text{bef}}<k_{i,\text{step }1}^{\text{bef}}$$

The correlation of Raman data observed in a biofilm of wild-type G. sulfurreducens did, however, not hold true when using an OmcS-deficient mutant. Although the current density was similar, the Raman dynamic response was significantly different than the modeled values, as seen in Fig. 7, which supports a different pathway or rate-limiting step. The Raman data showed a similar sharp decrease which was followed by a linear decline. The sharp decrease shows that there is a portion of the cytochromes that can quickly discharge to the electrode, seemingly at a higher rate than that in the G. sulfurreducens wild type. This correlates with the evidence that deletion of OmcS can actually increase biofilm conductivity, since it is the pili themselves that are responsible for the relatively high conductivity in G. sulfurreducens biofilms (7, 8). However, the slower linear oxidation implies that electron transfer is still hindered by the lack of OmcS.

One possible hypothesis is that the transfer of electrons to the outer cell membrane is hindered by the lack of OmcS. It has been previously proposed that OmcS might act as a conduit between cytochromes, meaning that a mutant lacking OmcS would require a different mechanism for EET (7). This implies that the rate of electron transfer to the outer membrane cytochromes, and hence to the extracellular matrix, might be hindered by the removal of OmcS, reducing the rate of oxidation of the intracellular cytochromes. The linear nature of the decline implies a pseudo-zero-order reaction; i.e., it is not significantly affected by the concentration of the reduced mediators. This could possibly be due to a limited quantity of a mediator being available in the alternative pathway, causing it to become saturated, resulting in a constant rate based on the total mediator available. If the limited mediator is identified, overexpression of it could possibly lead to a significant rate increase.

**FIG 9:**
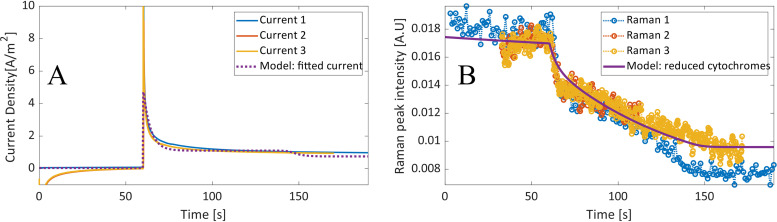
The fitted response of a modified model of the current density per electrode surface area (A) and Raman peak intensity (B) of an OmcS biofilm during a step change of poised voltage (from −251 mV to 649 mV).

In an attempt to model the OmcS-deficient strain, the IM_red_ oxidation rate equation was changed to be zeroth order with regard to [IM_red_] and [Omc_ox_]. This was divided by an exponential term in order to decrease the rate once an equilibrium point is reached. This resulted in a significantly better fit, as seen in Fig. 9. This empirical approach can be justified since zeroth-order reactions are common when the catalyst/enzyme is saturated.

(2) $$\frac{d\left[{\text{IM}}_{\text{red}}\right]}{\mathrm{dt}}=k_{\text{Ac}}\left[{\text{IM}}_{\text{ox}}\right]-\frac{k_{\text{Omc}}}{1+\exp\left(-\left[{\text{IM}}_{\text{red}}\right]+1,500\right)}$$

The results show that *in situ* resonance Raman microscopy and chronoamperometry can be effectively used, together with a dynamic model, to evaluate the dynamics of EET in a biofilm, as well as to show the impact of genetic changes on the dynamics. A standard model was capable of effectively estimating rate constants and predicting the redox state of cytochromes in the wild type. The fact that this was not true when using the OmcS mutant strain further stresses that OmcS is an important part of the EET in G. sulfurreducens. This also shows how useful a separate verification method is whenever a system this complex is modeled, not relying simply on the current.

## MATERIALS AND METHODS

### Model development.

In order to model the current and mediator concentrations, the reaction equations were converted into ordinary differential equations (ODEs) assuming elementary reactions. Two assumptions were made based on the characteristics of G. sulfurreducens biofilms. First, the rate of reduction of the internal mediators due to the reduction of acetate is a function not of acetate concentration but of only [IM_ox_]. Second, because of the high biofilm conductivity, the rate constant of the electron transfer through the biofilm (*k*_cond_) was assumed to be significantly higher than the rate of the electron transfer to the anode (*k*_cond_ ≫ *k_i_*) as well as the rate of electron transfer to ${\text{Omc}}_{\text{ox}}^{d=d_{0}}$ (*k*_cond_ ≫ *k*_Omc_). The change in concentration of the reduced extracellular mediators can be described by

(3) $$\frac{d\left[{\text{Omc}}_{\text{red}}^{d=d_{0}}\right]}{\mathrm{dt}}=-k_{\text{cond}}\left[{\text{Omc}}_{\text{ox}}^{d=0}\right]\left[{\text{Omc}}_{\text{red}}^{d=d_{0}}\right]+k_{\text{Omc}}\left[{\text{IM}}_{\text{red}}\right]\left[{\text{Omc}}_{\text{ox}}^{d=d_{0}}\right]$$

(4) $$\frac{d\left[{\text{Omc}}_{\text{red}}^{d=0}\right]}{\mathrm{dt}}=+k_{\text{cond}}\left[{\text{Omc}}_{\text{ox}}^{d=0}\right]\left[{\text{Omc}}_{\text{red}}^{d=d_{0}}\right]-k_{i}^{\text{*}}\left[{\text{Omc}}_{\text{red}}^{d=0}\right]$$

and be simplified to equation 3 + equation 4:

(5) $$\begin{array}{l} \frac{d\left[{\text{Omc}}_{\text{red}}^{d=d_{0}}\right]}{\mathrm{dt}}+\frac{d\left[{\text{Omc}}_{\text{red}}^{d=0}\right]}{\mathrm{dt}}\\ =k_{\text{Omc}}\left[{\text{IM}}_{\text{red}}\right]\left[{\text{Omc}}_{\text{ox}}^{d=d_{0}}\right]-k_{i}^{*}\left[{\text{Omc}}_{\text{red}}^{d=0}\right] \end{array}$$

If *k*_cond_ is sufficiently high, and the location of the reduced cytochrome does not affect the rate, the equation can be simplified to equation 6:

(6) $$\frac{d\left[{\text{Omc}}_{\text{red}}\right]}{\mathrm{dt}}=k_{\text{Omc}}\left[{\text{IM}}_{\text{red}}\right]\left[{\text{Omc}}_{\text{ox}}\right]-k_{i}^{\text{*}}\left[{\text{Omc}}_{\text{red}}\right]$$

The change in concentration of the reduced intracellular mediators can be described by

(7) $$\frac{d\left[{\text{IM}}_{\text{red}}\right]}{\mathrm{dt}}=k_{\text{Ac}}\left[{\text{IM}}_{\text{ox}}\right]-k_{\text{Omc}}\left[{\text{IM}}_{\text{red}}\right]\left[{\text{Omc}}_{\text{ox}}\right]$$

In order to limit the number of variables needed to be solved in order to fit the ODEs, the rate constant *k_i_* in equation 6 was fitted as the rate before a change in poised potential ($k_{i}^{\text{bef}}$), and after the change ($k_{i}^{\text{aft}}$), instead of fitting *k*^0^, α, and *E*^0^ from the Butler-Volmer equation.

(8) $$k_{i}^{*}=\{\begin{array}{c} k_{i}^{\text{bef}},t<t_{\text{switch}}\\ k_{i}^{\text{aft}},t\geq t_{\text{switch}} \end{array}$$

This results in a set of ODEs with six variables that need to be determined, namely, $k_{\text{Ac}},k_{\text{Omc}},k_{i}^{\text{bef}}\text{, and }k_{i}^{\text{aft}},$ as well as the total concentration of Omc and IM ([Omc]^tot^ and [IM]^tot^, respectively), described by equations 9 and 10:

(9) $${\left[\text{Omc}\right]}^{\text{tot}}\text{ = }\left[{\text{Omc}}_{\text{ox}}\right]\text{ + }\left[{\text{Omc}}_{\text{red}}\right]$$

(10) $${\left[\text{IM}\right]}^{\text{tot}}\text{ = }\left[{\text{IM}}_{\text{ox}}\right]\text{+ }\left[{\text{IM}}_{\text{red}}\right]$$

Lastly, the current density was calculated (equation 11) by multiplying the rate of electron transfer with the Faraday constant and dividing by the surface area of the electrode:

(11) $$\text{i}=F(k_{i}C_{\text{Omc}}^{\text{red}})\frac{1}{A}$$

### Preparation of measurement cells.

A two-chamber stack reactor built for online microscopy was used; details can be found in reference 18. The two chambers were separated using a proton exchange membrane (Nafion N117); both the cathode and anode were solid graphite electrodes (70 by 22 × 10 mm, with an exposed area of 3.3 × 10^−3^ m^2^), with Ag/AgCl reference electrode in the anode chamber. Silicone inserts were added on the sides of the graphite electrode in order to direct the flow across the face of the electrode.

### Microbes, media, and inoculation.

G. sulfurreducens strain PCA (ATCC 51573, DSMZ 12127) as well as a modified version of the strain (ΔOmcS), obtained from Ashley Franks, La Trobe University, Bundoora, Australia, was used in all studies. The ΔOmcS strain has a gene for the outer membrane cytochrome S removed (25).

All G. sulfurreducens inocula were grown in modified freshwater medium, as discussed in previous work (18), with acetate (10 mM) and fumarate (40 mM) as the electron donor and acceptor, respectively (26).

The microbial fuel cell stack used for Raman measurements were started in batch, using freshwater medium containing 20 mM acetate and 40 mM fumarate. The cathode chamber of the cell contained the same medium but without acetate and fumarate. The liquid in both chambers of the cell was recirculated to sparged bottles (20:80 CO_2_ to N_2_), using a peristaltic pump. The cells were poised at 300 mV (using a MultiEmStat+ potentiostat; PalmSens, Netherlands), and the anode chamber was inoculated with 10% inoculum as previously described (18, 27). Once sufficient current was established (>2 mA), the anode chamber’s recirculation bottle was removed and the anode chamber was fed continuously using the freshwater medium containing 10 mM acetate and no fumarate at a flow rate of approximately 0.5 ml min^−1^. This was maintained until a visible biofilm formed (2 to 4 weeks).

### Chronoamperometry and Raman measurements.

For dynamic measurements, a two-step chronoamperometry setup was used, with the anode initially poised at a low, constant potential versus a reference electrode, for 60 or 120 s, so that a steady state could be achieved, after which the voltage was stepped up to a higher value until a steady current was achieved. Two step change profiles were chosen, one with an initial value of −101 mV versus an SHE and then stepping up to 499 mV and the other with an initial value of −101 mV versus an SHE and then stepping up to 649 mV. The two minimum poising values were chosen to be above and below the formal redox potentials of OmcS and OmcZ, at −212mV and −220 mV, respectively (4, 23).

For Raman measurements, the cells were moved to the microscope, along with peristaltic pumps and anaerobic media, so that a steady state could be maintained. Resonance Raman spectra were collected, using an inverted microscope (Olympus IX71; Olympus, Tokyo, Japan), by choosing a 532-nm excitation diode-pumped solid-state (DPPS) laser (Altechna, Lithuania) in order to obtain a higher signal strength coupled to a Raman spectrometer, Shamrock 303i (Andor Technology, Belfast, UK). The laser operated at approximately 15 mW prior to the microscope objective, with a 1-s integration time and 5 accumulations. Raman measurements were taken from 30 to 60 s before the step change in poising voltage of the multistep chronoamperometry. The cosmic rays were removed and a baseline estimation and denoising filter was applied to the raw resonance Raman spectra (18, 28, 29). Of the bands that can be ascribed to the excitation of the heme groups of cytochrome *c* (747, 1,131, 1,317, and 1,587 cm^−1^), the peak at 747 cm^−1^ is the most intense and was therefore used to measure the oxidation state of the cytochromes (30–32). The peak was integrated over a fixed window of 22 cm^−1^ using MATLAB R2018 to obtain the area below the peak. Since the Raman data represent only a single spot of the biofilm, they cannot be quantitatively compared to the entire biofilm. Instead, the Raman data were normalized with regard to area under the curve, and the total reduced mediators was multiplied by an empirical factor for each set of experiments in order to align it with the Raman data.

A constant voltage measurement was performed in order to determine if the signal strength decreases over time, due to localized damage caused by the laser beam, and although there was some decline over a longer period, the change was deemed insignificant due to the relatively short measurement durations (±180 s).
